# Validation of a risk score to differentiate autoimmune and viral encephalitis: a Nationwide Cohort Study in Denmark

**DOI:** 10.1007/s00415-024-12392-3

**Published:** 2024-05-18

**Authors:** Lasse Fjordside, Mette Scheller Nissen, Anna Maria Florescu, Merete Storgaard, Lykke Larsen, Lothar Wiese, Hans Rudolf von Lüttichau, Micha Phill Grønholm Jepsen, Birgitte Rønde Hansen, Christian Østergaard Andersen, Jacob Bodilsen, Henrik Nielsen, Morten Blaabjerg, Anne-Mette Lebech, Helene Mens

**Affiliations:** 1 Department of Infectious Diseases, Copenhagen University Hospital, Rigshospitalet, Copenhagen, Denmark; 2 Department of Neurology, Odense University Hospital, Odense, Denmark; 3 Department of Infectious Diseases, Aarhus University Hospital, Aarhus, Denmark; 4 Department of Infectious Diseases, Odense University Hospital, Odense, Denmark; 5 Department of Infectious Diseases, Sjællands University Hospital, Roskilde, Denmark; 6 Department of Infectious Diseases, Herlev and Gentofte Hospital, Copenhagen, Denmark; 7 Department of Pulmonology and Infectious Diseases, Nordsjællands University Hospital, Hillerød, Denmark; 8 Department of Infectious Diseases, Hvidovre University Hospital, Copenhagen, Denmark; 9 Department for Infectious Disease Preparedness, Statens Serum Institut, Copenhagen, Denmark; 10 Department of Infectious Diseases, Aalborg University Hospital, Aalborg, Denmark; 11 Department of Clinical Medicine, Aalborg University, Aalborg, Denmark; 12 Institute of Clinical Medicine, University of Copenhagen, Copenhagen, Denmark

**Keywords:** Encephalitis, Autoimmune encephalitis, Viral encephalitis, Risk score, Validation

## Abstract

**Background:**

A score to differentiate autoimmune (AE) and viral encephalitis (VE) early upon admission has recently been developed but needed external validation. The objective of this study was to evaluate the performance of the score in a larger and more diagnostically diverse patient cohort.

**Methods:**

We conducted a retrospective nationwide and population-based cohort study including all adults with encephalitis of definite viral (2015–2022) or autoimmune aetiology (2009–2022) in Denmark. Variables included in the score-model were extracted from patient records and individual risk scores were assessed. The performance of the score was assessed by receiver-operating characteristics (ROC) curve analyses and calculation of the area under the curve (AUC).

**Results:**

A total of 496 patients with encephalitis [AE *n* = 90, VE *n* = 287 and presumed infectious encephalitis (PIE) *n* = 119] were included in the study. The score was highly accurate in predicting cases of AE reaching an AUC of 0.94 (95% CI 0.92–0.97). Having a score ≥ 3 predicted AE with a PPV of 87% and an NPV of 91%. The risk score was found to perform well across aetiological subgroups and applied to the PIE cohort resulted in an AUC of 0.88 (95% CI 0.84–0.93).

**Conclusion:**

The excellent performance of the score as reported in the development study was confirmed in this significantly larger and more diverse cohort of patients with encephalitis in Denmark. These results should prompt further prospective testing with wider inclusion criteria.

**Supplementary Information:**

The online version contains supplementary material available at 10.1007/s00415-024-12392-3.

## Introduction

Encephalitis is a rare but severe clinical condition with a substantial mortality and a high risk of neurological sequelae [1–3]. Early treatment initiation is paramount for optimized patient outcomes, but determining the aetiology is often challenging [4–6]. Viral aetiologies can be difficult to detect by conventional molecular-based methods. Thus, a pathogen is not identified in 35–60% of cases clinically considered as viral encephalitis (VE) [7, 8].

Additionally, a definite diagnosis of autoimmune encephalitis (AE) requires detection of disease-specific neuronal autoantibodies which is often logistically challenging and time-consuming.

To facilitate the clinical recognition of AE and to support early initiation of immunotherapy, *Graus* and colleagues proposed a set of diagnostic criteria in 2016 [9].

However, the time to initiation of anti-inflammatory treatment in patients with AE is still significantly delayed [10].

To overcome these challenges, *Granillo* and colleagues recently developed a risk score that accurately predicts cases of AE [11].

We believed that the risk score could be a valuable clinical tool in differentiating AE and VE, reduce delays in relevant treatment initiation, and thereby improve outcomes for patients with encephalitis. However, external validation on a larger and more diagnostically diverse population is needed.

We therefore aimed to test the performance of the score on a large nationwide cohort of patients with AE and VE in Denmark.

## Materials and methods

### Design, study population and setting

The study was carried out as a retrospective nationwide cohort study including all patients with a documented episode of definite viral or autoimmune encephalitis in Denmark from 2009 to 2023. A cohort of 119 patients with presumed infectious encephalitis (PIE) but no verified pathogen was additionally identified and assessed with the risk score.

All patients were identified from two nationwide databases:

1. Danish Study Group for Infections of the Brain (DASGIB) database: this database includes all reported cases of infections in the central nervous system of adults in Denmark from 2015 to present.
2. The National Database for Autoimmune Encephalitis (NDAE): this database includes all reported cases of autoimmune encephalitis in Denmark from 2009 to present.

The study was initiated in January 2023 by the Department of Infectious Diseases at Copenhagen University Hospital, Rigshospitalet, Copenhagen, Denmark. Basic demographic and diagnostic information was obtained from the databases. Additionally, electronical patient records were reviewed by four different investigators to ensure the fulfillment of inclusion criteria of all cases as well as to extract the additional and necessary clinical and paraclinical information for performing the individual score-assessment.

Data extraction was completed by August 2023. Results are reported in accordance with the STARD-2015 guidelines on reporting of diagnostic accuracy studies [12].

### Participants

A total of 641 patients in a consecutive series were screened for inclusion and 377 patients with AE (*n* = 90) and VE (*n* = 287) were enrolled in the study. We additionally assessed the score performance on a group of 119 patients with presumed infectious encephalitis, but no verified infectious or autoimmune aetiology (PIE). Inclusion criteria were identical with those in development study [11] (supplementary appendix I).

### Case definitions

*VE*: Cases of VE were identified from the DASGIB Database. All enrolled cases of VE fulfilled the International Encephalitis Consortium (IEC) criteria for confirmed encephalitis and had a viral neurotropic pathogen verified in cerebrospinal fluid (CSF), either by direct PCR or with a positive pathogen-specific intrathecal antibody test [13] (supplementary appendix I).

*AE*: Cases of AE were identified from the NDAE. All enrolled cases of AE fulfilled the consensus criteria for AE and had a positive test for a disease-specific autoantibody in CSF and/or blood [9].

*PIE*: Cases of PIE were identified from the DASGIB Database. All cases of PIE fulfilled the IEC criteria for probable encephalitis and had a final clinical diagnosis of ‘infectious encephalitis of unknown aetiology’. The diagnosis was established by an infectious disease specialist based on clinical presentation, paraclinical findings, and treatment response, but without a verified pathogen. All had a negative PCR for neurotropic pathogens, and none had a positive test for neuronal autoantibodies.

### Index test

In this validation study, we aimed to assess the accuracy of the risk score for AE developed by *Granillo *et al.[11] (Table 1). The score ranges from 0 to 4, with higher scores indicating a higher risk of AE.

**Table 1:** The risk score for autoimmune encephalitis and risk categories based on accumulated score values as developed by *Granillo* and colleagues

Score elements	Points	
	Score element	
	Present = 1	Absent = 0
Charlson comorbidity index < 2	0–1	
Subacute (6–30 d) to chronic (> 30 d) onset	0–1	
Psychiatric and/or memory complaints	0–1	
Absence of robust inflammation in CSF (WBC < 50/μL and protein < 50 mg/dL)	0–1	
Accumulated score	0–4	
Risk categories^a^		
Low risk	0–1	
Intermediate risk	2–3	
High risk	4	

*CSF* cerebrospinal fluid, *WBC* white blood cell count

^a^Based on accumulated score values

The Charlson Comorbidity Index (CCI) was established by information on age and comorbidities documented at admission[14]. Psychiatric complaints were defined as symptoms of true psychiatric character (hallucinations, delusions, mania, depression, or severe anxiety). Memory complaints were defined as new onset or exacerbated memory deficits as part of the acute disease. The terms “confusion” or “disorientation” were not considered a memory or a psychiatric complaint.

Onset of encephalitis was defined as the onset of new neurological symptoms and the duration of symptoms was defined as the number of days from onset to first hospital contact. The number of white blood cells (WBC) and the level of protein in CSF were recorded from the lumbar puncture performed at the primary admission for encephalitis.

If standard test results were not available or assessment of the risk score was not possible due to missing data, cases were excluded.

### Statistical methods

Data were analyzed using ‘R Studio’ 4.2.2 software [15]. The accuracy of the risk score for identification of AE cases was determined by calculating receiver-operating characteristics curve (ROC) and the area under the ROC curve (AUC) using the ‘pROC’ package in ‘R’. Confidence intervals for the AUC estimates were calculated by performing 1000 resampling-bootstraps using the ‘boot’ package in ‘R’. Chi-squared and Mann–Whitney U tests were performed to calculate the statistical difference between the score results of the different diagnostic groups. We chose a confidence interval of 95% and considered p values < 0.05 significant.

### Ethical statement

The DASGIB cohort was approved by the Danish Board of Health (3–3013-2579/1 and 3–3012-3168/1) and The Danish Data Protection Agency (2012–58-0018). Additional permission to access patient files was approved by the Regional Office for Journal Data (R-23004082). Approval for the AE cohort was similarly obtained from the Danish Data Protection Agency (3–3013-3124/1) and the Danish Board of Health and granted with a waiver for individual consent.

## Results

### Descriptive data

#### The AE and VE cohorts

**Fig. 1:**
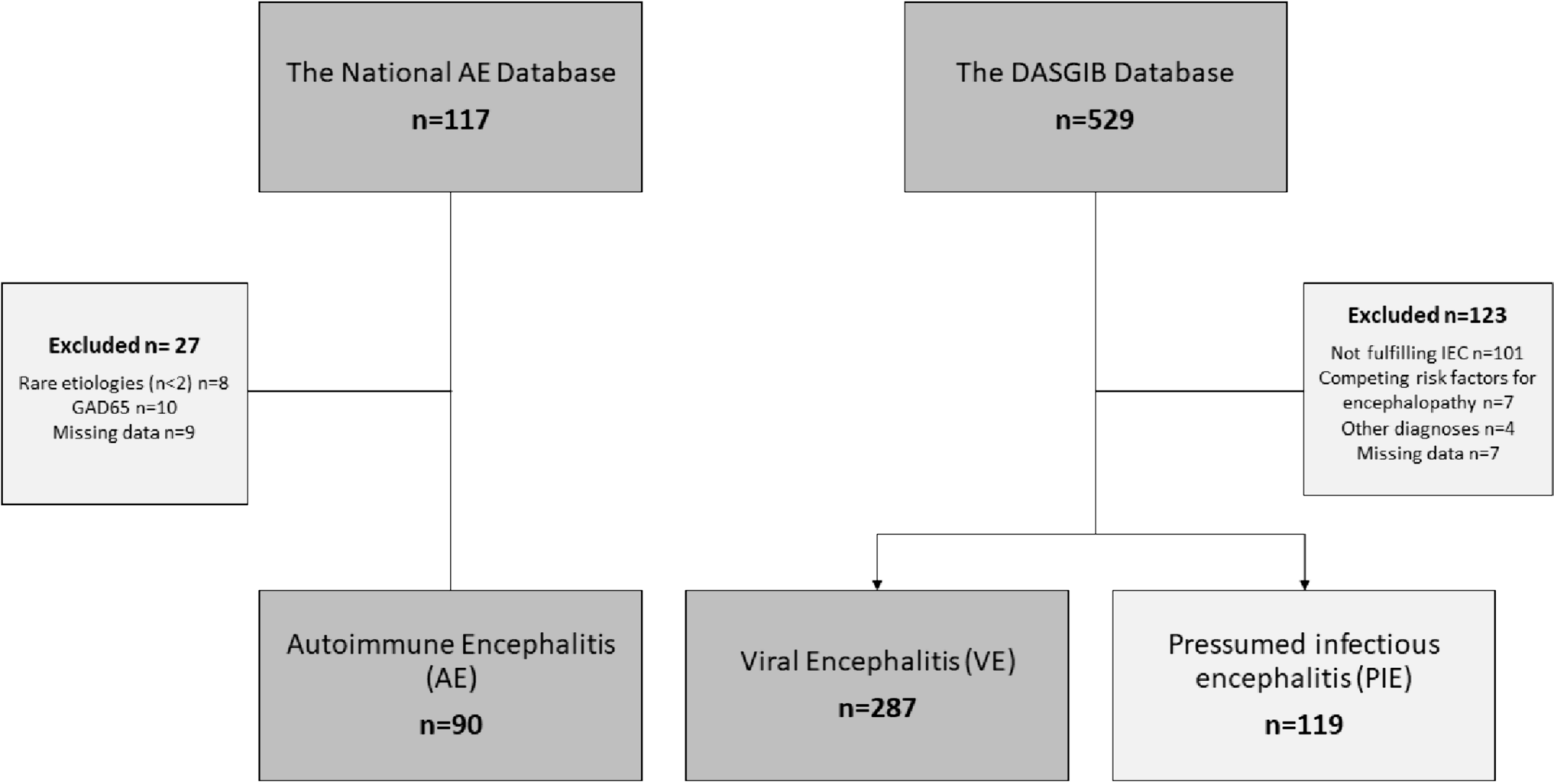
Flow diagram of the inclusion process

Ninety patients with AE and 287 patients with VE fulfilled all inclusion criteria (Fig. 1). Patients with AE were significantly younger than patients with VE (mean of 45 with SD of 23.6 versus a mean of 68, with SD of 16.5, p < 0.001), but had a similar sex distribution (52% females) (Table 2). The largest diagnostic subgroup in the AE cohort was N-methyl-D-aspartate receptor (NMDAR) encephalitis comprising over half of the AE cohort. This group included six patients who had their NMDAR encephalitis after an episode of HSV1-encephalitis. The remainders had other aetiologies associated with higher age and comorbidity such as leucine-rich glioma-inactivated-1 (LGI1), γ-aminobutyric-acid-type-B (GABA-B), and alfa-amino-3-hydroxy-5-methyl-4-isoxazolpropionsyre-receptor (AMPAR). The VE cohort was dominated by viruses from the Herpes family (92%), whereas the remaining 8% were made up by flavi- and retroviruses.

**Table 2:** Baseline characteristics of patients identified for inclusion in the autoimmune- and the viral encephalitis cohorts for validation of the risk score for AE

	Autoimmune, *n* = 90	Viral, *n* = 287	Presumed infectious, *n* = 119	*P* values^a^
Age, mean (SD)	45 (23.6)	68 (16.5)	57(19.7)	< 0.001^a^
Female, *n* (%)	47 (52)	149 (52)	51(43)	> 0.9^a^
Diagnoses, *n* (%)				
Autoimmune				
NMDAR	58 (64)	–	–	–
LGI1	23 (26)	–	–	–
GABA-B	6 (7)	–	–	–
AMPAR	3 (3)	–	–	–
Viral				
HSV1	–	126 (44)	–	–
VZV	–	122 (43)	–	–
TBE	–	20 (7)	–	–
HSV2	–	12 (4)	–	–
HIV	–	5 (2)	–	–
EBV	–	2 (1)	–	–

^a^*p* values are on the difference between the AE and the VE cohorts only

*SD* standard deviation, *NMDAR* *N*-methyl-d-aspartate-receptor, *LGI1* leucine-rich glioma-inactivated 1, *GABA-B* γ-aminobutyric acid type B, *AMPAR* alpha-amino-3-hydroxy-5-methyl-4-isoxazolepropionic acid receptor, *HSV1* herpes simplex virus 1, *VZV* varicella zoster virus, *TBE* tick-borne encephalitis, *HSV2* herpes simplex virus 2, *HIV* human immunodeficiency virus, *EBV* Epstein-Barr virus

#### The PIE cohort

The PIE cohort consisted of 119 individuals. This group of patients were younger than patients in the VE cohort with a mean age of 57 years and an SD of 19.7 versus mean of 68 years, with SD of 16.5, (p < 0.001) and included a smaller proportion of females [43% vs 52% (p < 0.001)] (Table 2).

### Main results

**Fig. 2:**
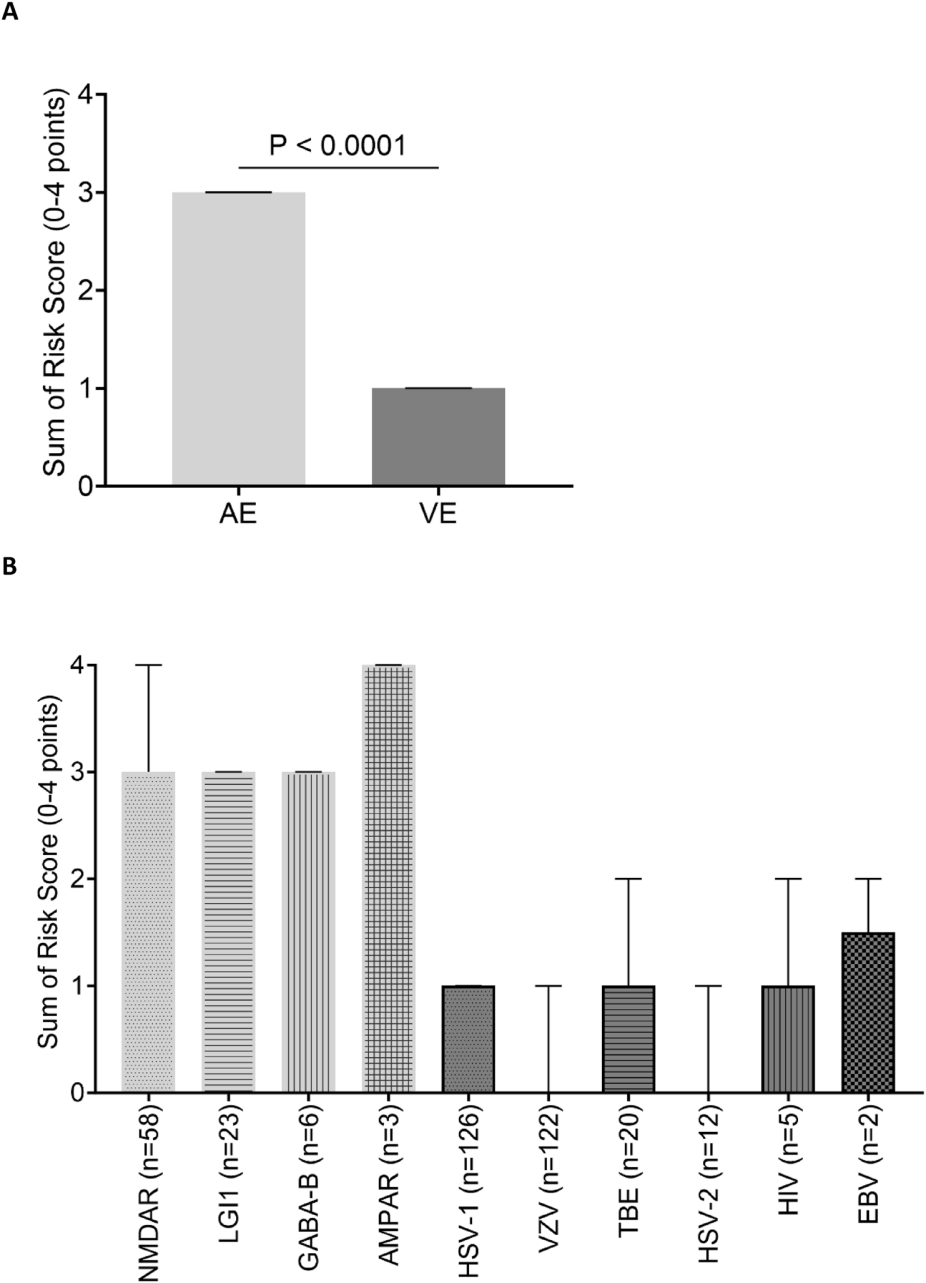
Bar charts comparing median score values and interquartile ranges of the AE and VE cohorts. **A** Compares the median score values between the AE and the VE cohort and **B** depicts the median score values by aetiological subgroups of the AE (light gray) and VE (dark gray), with fill patterns indicating individual diagnosis

**Fig. 3:**
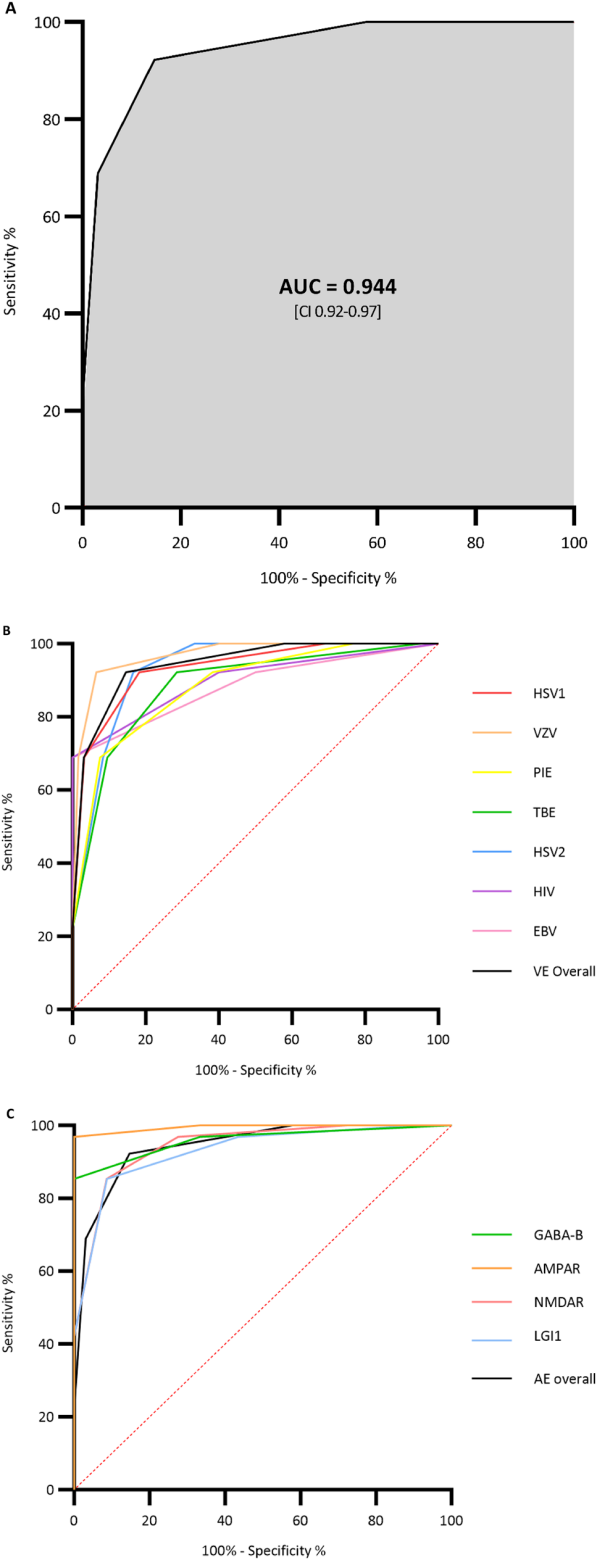
Receiver operating characteristics (ROC) curves and area under the curve (AUC) for the performance of the risk score to differentiate the AE from the VE cohort (**A**) and ROC curves for the comparison of the score performance for each of the different viral aetiological subgroups (**B**) as well as for each of the different autoimmune aetiological subgroups (**C**)

Risk-score values were significantly higher in the AE cohort compared to the VE cohort [median of 3 (IQR 2–3) vs median of 1 (IQR 0–1), p < 0.001)] (Fig. 2A). Score values were similar across aetiological subgroups in the AE cohort and only showed slight variations in the VE cohort (Fig. 2B). The risk score reached an AUC of 0.94 (95% CI 0.92–0.97) for predicting AE (Fig. 3A). The score performance in the VE cohort varied across aetiologies from AUC = 0.98 (95% CI 0.95–0.99, VZV) to AUC = 0.88 (95% CI 0.80–0.96, TBEV) (Fig. 3B). The score performance was high and only showed marginal variation across AE aetiologies (Fig. 3C). A score ≥ 3 resulted in a PPV of 87% and an NPV of 91% (Table 3). Patients < 18 years of age in the AE cohort (n = 13), had significantly higher score values than those ≥ 18 years (n = 77) [medians and (IQR): < 18 years: 3 (2,3) vs. ≥ 18 years: 3(3,4) p value < 0,001].

The performance of the score in the PIE cohort (Fig. 3B) was consistently high yet slightly inferior to the overall VE cohort reaching an AUC of 0.88 (95% CI 0.84–0.93), corresponding to a sensitivity of 69% and specificity of 93% with a score of ≥ 3 (supplementary appendix III). Slightly higher score values were observed for patients in the PIE cohort compared to the VE cohort, but the values were significantly lower than those in the AE cohort. The PIE cohort diverted most from the VE cohort by having a higher proportion of patients with a CCI < 2 (Table 4).

**Table 3:** Sensitivity, specificity, positive predictive value (PPV), and negative predictive value (NPV) for the prediction of AE for the different score values

Score	Sensitivity	Specificity	PPV	NPV
0	100%	0%	24%	0%
1	100%	56%	35%	100%
2	92%	85%	66%	97%
3	69%	97%	87%	91%
4	23%	100%	100%	81%

**Table 4:** Score distribution in the autoimmune, viral, and presumed infectious encephalitis cohorts

	Autoimmune, *n* = 90	Viral, *n* = 287	Presumed infectious, *n* = 119	*p* values^a^
Score elements, *n* (%)				
CCI < 2	50 (56)	47 (16)	51(43)	0.005
Subacute onset^a^	67 (74)	33 (11)	29(24)	< 0.001
Psychiatric and/or memory complaints	87 (97)	48 (17)	33(28)	< 0.001
Absence of robust inflammation in CSF^b^	52 (58)	90 (31)	33(28)	0.004
Sum of score, *n* (%)				
0	0 (0)	121 (42)	28(24)	< 0.001
1	7 (8)	124 (43)	45(38)	< 0.001
2	21(23)	33 (11)	37(31)	0.045
3	41 (46)	9 (3)	9(8)	< 0.001
4	21 (23)	0 (0)	0(0)	< 0.001
Risk categories, *n* (%)				
Low (0–1)	7 (8)	245 (85)	73(61)	< 0.001
Intermediate (2–3)	62 (69)	42 (15)	46(39)	–
High (4)	21 (23)	0 (0)	0(0)	< 0.001

*CCI* Charlson Comorbidity Index, *CSF* cerebrospinal fluid

^a^Onset of neurological symptoms > 6 days prior to admission

^b^Defined as white blood cell count (WBC) < 50/μL and protein < 50 mg/dL

^c^*p* Values are calculated on the difference between the Autoimmune and the Viral cohort

### Score distribution

**Fig. 4:**
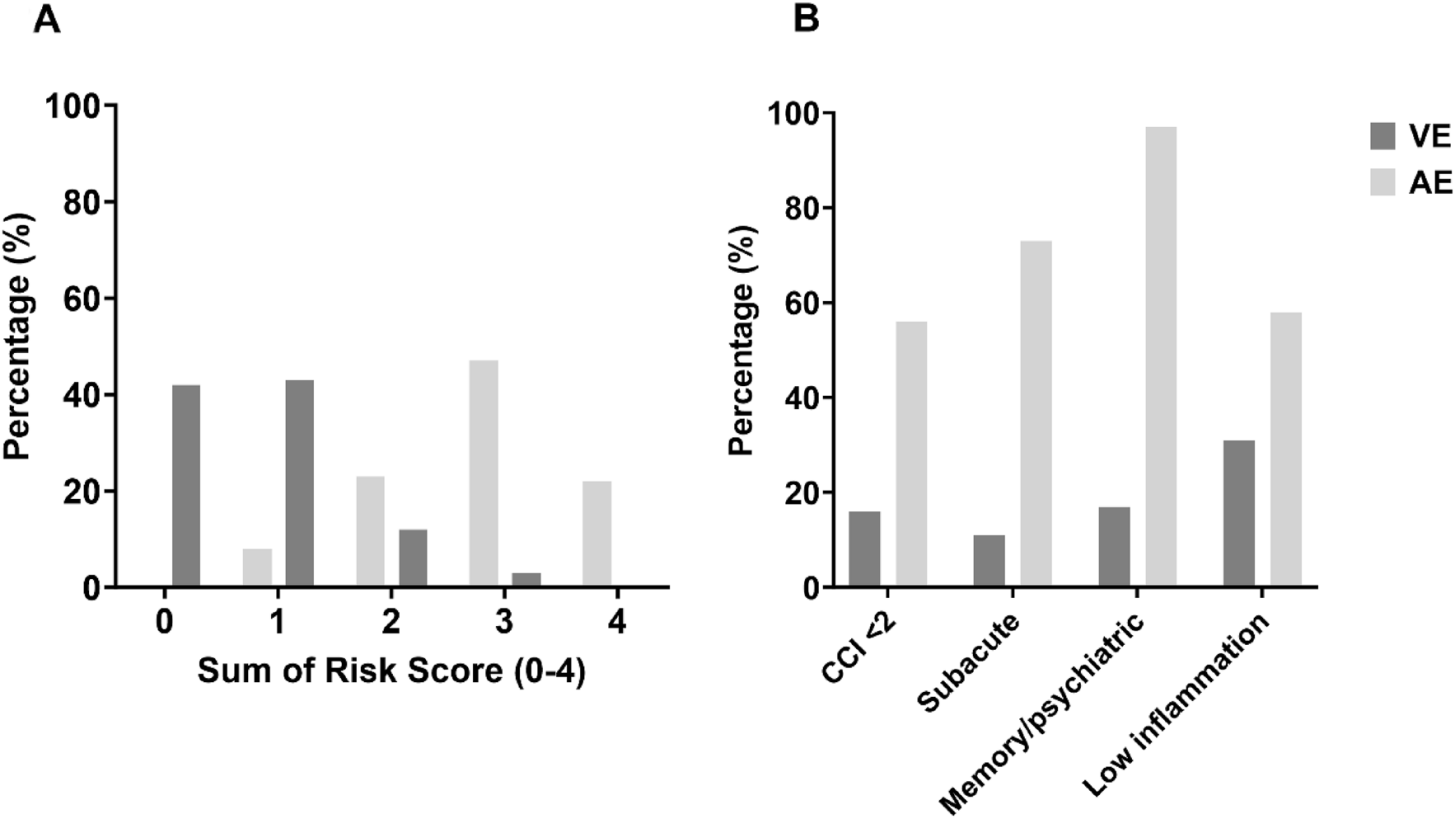
Bar chart comparing the score distribution in the AE and the VE cohorts by accumulated score values **A** and score elements **B**

The score distribution in the AE, VE, and PIE cohorts (Table 4 and Fig. 4A) followed a Gaussian-like pattern with clear differences in approximated means. Based on score values, 8% of patients in the AE cohort and 85% of patients in the VE cohort were classified as ‘low AE risk’ (score value 0–1) (p < 0.001), whereas 23% in the AE cohort and 0% in the VE cohort were classified as ‘high AE risk’ (score value 4) (p < 0.001) (Table 4).

### Score distribution by score elements

**Fig. 5:**
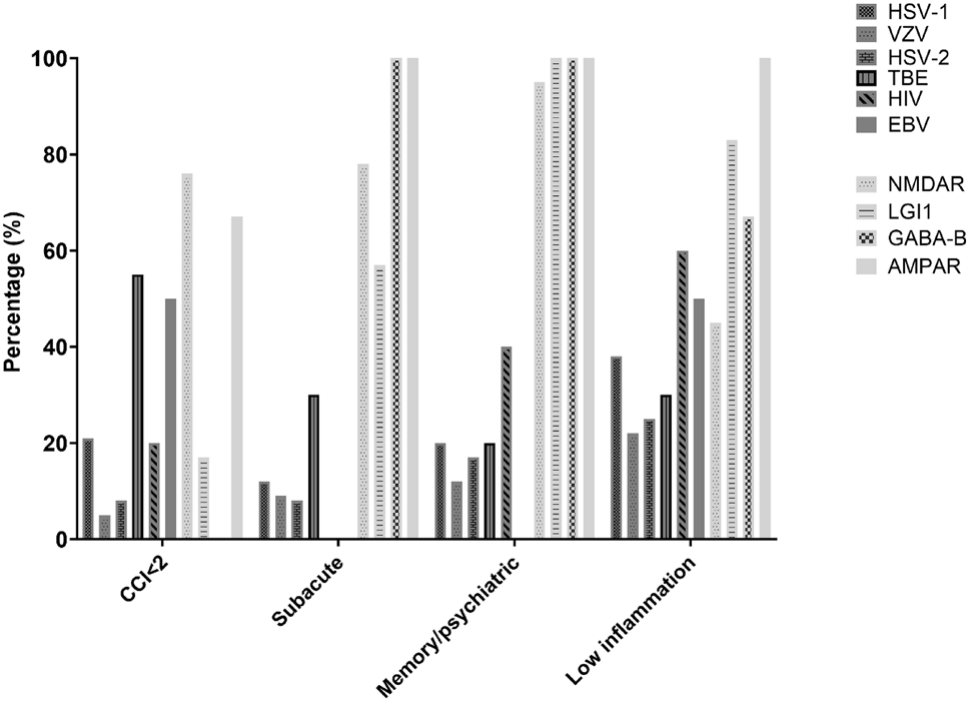
Bar chart comparing the score distribution by score elements for each of the diagnostic subgroups. HSV1 herpes simplex virus 1, VZV varicella zoster virus, TBE tick-borne encephalitis, HSV2 herpes simplex virus 2, HIV human immunodeficiency virus, EBV Epstein-Barr virus, NMDAR N-methyl-d-aspartate-receptor, LGI1 leucine-rich glioma-inactivated 1, GABA-B γ-aminobutyric acid type B, AMPAR alpha-amino-3-hydroxy-5-methyl-4-isoxazolepropionic acid receptor, CCI

For all score elements, patients in the AE cohort had the highest fulfillment proportion (Table 4 and Fig. 4B). The relative difference in the proportion of patients with ‘subacute onset’ and ‘psychiatric/memory complaints’ was more pronounced compared to the remaining score variables (Fig. 5).

The NMDAR and AMPAR subgroups had less comorbidities than patients in the LGI1 and GABA-B subgroups and patients in the NMDAR subgroup more frequently had robust CSF inflammation than patients in the other subgroups. In the VE cohort, patients in the TBEV subgroup had less comorbidities and a higher frequency of subacute disease presentation. Patients in the HSV-2 and VZV subgroups had more comorbidities and a higher likelihood of robust CSF inflammation compared to patients in the HSV-1 group (Fig. 5).

## Discussion

### Main results

The score reached an AUC of 0.94 for the prediction of AE in this Danish cohort of 377 encephalitis patients. A score ≥ 3 predicted AE with a PPV of 87% and an NPV of 91%.

### Comparing results with the development study

The score performance was comparable to what was found in the development study, even though patients in our cohorts were slightly older than those in the development study (mean of 62.5 years ± 20.8 versus mean of 50.6 years ± 19.7) but had a similar sex distribution with 52% females. The VE cohort in this study was dominated by herpes viruses, whereas the original study included an approximately equal number of cases with herpes- and flaviviruses.

### Minor variation in score performance across the aetiological subgroups

We found it reassuring that the performance of the score was not negatively affected by the higher age and CCI for patients in the LGI1 group and the higher rate of CSF inflammation in NMDAR.

While reaching high AUCs for all diagnostic subgroups included in this study, we did observe relatively better performance for herpes virus aetiologies compared to TBEV. This difference was driven by less comorbidity and a higher frequency of subacute presentation among patients in the TBEV group. Although the inter-aetiological variation in score performance in the VE cohort does raise a question of the translational potential of the score to settings with significantly different aetiological patterns, we expect that the score will perform well in a European setting where aetiologies for AE and VE are comparable[10, 16, 17]

### The risk score performed well in patients with presumed infectious encephalitis

Patients in the PIE group were younger, more often males and had a lower but still acceptable score performance compared to the VE cohort (AUC = 0.88 versus 0.94) translating into a sensitivity of 69% and specificity of 93% with a score of ≥ 3. The score distribution of the PIE cohort deviated most significantly from the VE cohort by a higher proportion with a CCI < 2, likely corresponding to the lower mean age. The somewhat inferior performance of the score in the PIE cohort could potentially be caused by undiagnosed or sero-negative cases of AE. Alternatively undiagnosed atypical infectious aetiologies with differing clinical and paraclinical presentations (e.g., the TBEV group) could also contribute to an impaired performance of the score.

### Presence of psychiatric symptoms and subacute presentation most clearly distinguished AE and VE

AE cases differed most significantly from VE cases on the longer time from symptom onset to presentation and on the presence of psychiatric- and/or memory complaints, suggesting that these parameters are essential to differentiate between the two populations. This reflects findings in the previous studies[18, 19]. Also worth noticing is that these variables are likely more robust to future epidemiological transitions. As an example, the CCI item could be affected by a change toward older mean ages in future AE populations. Likewise, the absence of robust inflammation in CSF could be affected by an increasing proportion of immunosuppressed individuals in future VE populations [20].

### Should we modify the score?

We did not include additional parameters than those reported in the development study, since this was solely intended to be a validation study. However, it would be interesting to compose and test a modified risk score for AE by adding some of the parameters highlighted in recent observational studies where absence of fever, absence of hyponatremia, and the presence of movement disorders were identified as significant risk factors for AE [10, 19]. Some of these risk factors were also identified in the development study[11]. Inclusion of more variables could bring the secondary advantage of increasing the numeric value of the score and thereby potentially allowing for a more accurate risk stratification.

Even though the score could probably be improved by modifications, we believe that the consistently high performance in this external validation cohort should be sufficient to qualify the risk score for prospective testing.

## Strengths and limitations

The large study population, the diagnostic diversity in the AE cohort, and the well-validated cases are the main strengths of this study. The retrospective design represents the main limitation, primarily because it introduces a risk of assessment bias as previously described[21].

### Bias assessment

Only allowing inclusion of patients with verified aetiologies of AE and VE may create a risk of partial verification bias and disease spectrum bias as described by *Kennedy* et al. potentially causing an overestimation of sensitivities and specificities[22]. The unblinded-retrospective study design causes an inherent risk of assessment bias. In this study, the risk of assessment bias was primarily related to the registration of psychiatric symptoms. We aimed at minimizing this risk by establishing criteria for the registration of psychiatric symptoms that were followed by all assessors (see supplementary appendix II). Arguably a prospective study with wider inclusion criteria would be ideal for testing the real-life performance of the score; however, such investigation would need to be performed as an extensive multi-center study with a long inclusion period given the low incidence of encephalitis. We believe that our external validation study is an essential stepping-stone to such large-scale prospective testing.

### Clinical implications

We believe that the score developed by *Granillo* et al. can be a valuable clinical tool for AE-risk-stratification of patients with encephalitis early upon admission. Early identification of patients at high risk of AE can prompt earlier investigation for autoimmune aetiologies and thereby hopefully reduce the diagnostic delay. Additionally, the score could be used to support decisions of sustained antiviral treatment in PCR-negative patients with low risk scores for AE.

## Conclusion

The excellent performance of the risk score reported in the development study was confirmed in this large and diagnostically diverse Danish setting. We believe that these results justify large-scale prospective testing of the risk score with wider inclusion criteria to assess the real-life utility.

### Supplementary Information

Below is the link to the electronic supplementary material.

Supplementary file1 (DOCX 29 KB)

## Data Availability

Raw data from this study can not be made publicly available due to their patient-sensitive nature requirering collaboration agreements with explicit agreements on confidentiallity and restrictions on the use. However, R-code can be shared by corresponding author upon request.
