# Supplementary material for: Validation of a risk score to differentiate autoimmune and viral encephalitis: a Nationwide Cohort Study in Denmark
Source: J Neurol. 2024 May 18;271(8):4972–81. doi: 10.1007/s00415-024-12392-3 (PMC11319475; doi:10.1007/s00415-024-12392-3)
Supplement: Supplementary file 1 — Supplementary file1 (DOCX 29 KB) [file 415_2024_12392_MOESM1_ESM.docx]

**Supplementary appendix**

**I. Inclusion criteria**

All patients with **viral encephalitis** had to fulfil the 2013 International Encephalitis Consortium (IEC) criteria for encephalitis(1):

1. Patients presenting with altered mental status (defined as decreased or altered level of consciousness, lethargy, or personality change) lasting ≥24 h with no alternative cause identified.

**AND**

1. At least 3 of the following:

- Fever ≥38 °C (100.4 °F) within the 72 h before or after presentation.
- Seizures not fully attributable to a pre-existing seizure disorder.
- New onset of focal neurologic findings.
- CSF white blood cell (WBC) count ≥5 cells/mm3.
- Abnormality of brain parenchyma on neuroimaging suggestive of encephalitis that is either new from prior studies or appears acute in onset.
- Abnormality on electroencephalography that is consistent with encephalitis and not attributable to another cause.

**AND ADDITIONALLY**

1. A neurotropic viral pathogen in CSF verified by either PCR or pathogen-specific intrathecal antibody test.

All patients with **autoimmune encephalitis** had to fulfil the *Graus’* criteria for definite autoimmune encephalitis (all of the below)(2)**:**

1. Subacute onset (rapid progression of <3 months) of working memory deficits (short-term memory loss), altered mental status, or psychiatric symptoms.

**AND**

1. At least 1 of the following:
   - New focal central nervous system findings
   - Seizures not explained by a previously known seizure disorder
   - CSF pleocytosis (WBC count >5 cells/mm3)
   - Magnetic resonance imaging features suggestive of encephalitis.

**AND**

1. Exclusion of alternative causes

**AND ADDITIONALLY**

1. Presence of laboratory confirmation of antibodies against neuronal cell-surface or synaptic proteins.

**II. Case examples of the inclusion assessment**

**Example 1**: a 73- y/o woman presenting with sudden onset confusion and a fever. She had a monocytic pleocytosis and the PCR in CSF was positive for Herpes simplex virus 1 (HSV1), but there was no available description of EEG or MRI in the patient record. The final clinical diagnosis of this patient was HSV1-encephalitis and the patient responded well to treatment with acyclovir. After consulting a senior infectious disease specialist, it was decided to include this case in the study as a diagnosis of HSV1 encephalitis was deemed highly plausible.

**Example 2**: A 55 y/o male with excessive alcohol consumption presenting with a peripheral facial palsy, a vesicular rash around his left ear and a fever. Upon admission the patient was perceived as somewhat reluctant to further diagnostic investigations but not described as encephalopathic. He had a monocytic pleocytosis and the PCR in CSF was positive for Varicella zoster virus (VZV), the MRI of the brain was without signs of encephalitis and EEG was not performed. This individual was *not* enrolled because although fulfilling the minor criteria (fever, pleocytosis and new focal neurological deficit), we could not with certainty say that he met the major criteria of ‘altered mental status’

**III. Supplementary Table 1**: Sensitivity, specificity, positive predictive value (PPV) and negative predictive value (NPV) for the prediction of AE when using the PIE cohort instead of the VE cohort as non-case comparison.

| Score | Sensitivity | Specificity | PPV | NPV |
| --- | --- | --- | --- | --- |
| *0* | 100% | 0% | 43% | 0% |
| *1* | 100% | 22% | 49% | 100% |
| *2* | 92% | 60% | 63% | 91% |
| *3* | 69% | 93% | 87% | 80% |
| *4* | 23% | 100% | 100% | 64% |

1. Venkatesan A, Tunkel AR, Bloch KC, Lauring AS, Sejvar J, Bitnun A, et al. Case definitions, diagnostic algorithms, and priorities in encephalitis: Consensus statement of the international encephalitis consortium. Clin Infect Dis. 2013;57(8):1114–28.

2. Graus F, Titulaer MJ, Balu R, Benseler S, Bien CG, Cellucci T, et al. A clinical approach to diagnosis of autoimmune encephalitis. Vol. 15, The Lancet Neurology. 2016. p. 391–404.
